# Advances in infection-immunity mechanisms and molecular regulatory networks in severe pneumonia-associated lung injury

**DOI:** 10.3389/fimmu.2026.1702084

**Published:** 2026-03-17

**Authors:** Xuan Zhao, Jun Gao, Tianyi Wang, Qiongling Sun, Jing Yu, Wensen Pan

**Affiliations:** Second Department of Respiratory and Critical Care Medicine, The Second Hospital of Hebei Medical University, Shijiazhuang, China

**Keywords:** cytokine storm, infection-immunity, molecular regulatory network, severe pneumonia-associated lung injury, targeted intervention

## Abstract

Severe pneumonia-associated lung injury remains a critical focus in infection and immunology research due to its high lethality and complex immunopathology. Affected patients often present with cellular immune deficiency and an imbalanced interplay between pro-inflammatory and anti-inflammatory mediators, with infection-immunity disequilibrium driving disease progression. The initial infection triggers a defense-injury cascade: while inflammation facilitates pathogen clearance, persistent or excessive activation leads to progressive lung tissue damage. Recent advances have deepened our understanding of immune mechanisms and inflammatory regulation; however, key challenges persist in clinical translation. Here, we synthesize current evidence on the immunopathology of severe pneumonia-associated lung injury, dissect the hierarchical architecture of its molecular regulatory networks, and critically appraise existing therapeutic strategies and their limitations. We propose a translational framework—encompassing mechanistic research, network modelling, and precision intervention—to guide stage-specific regulation and targeted therapy. Finally, we outline the major challenges impeding the clinical application of basic immunological discoveries and highlight future directions for improving patient outcomes.

## 1 Introduction

Severe pneumonia represents a major challenge in respiratory critical care, arising from diverse pathogens including bacteria and viruses. Its pathogenesis is characterized by persistent pathogen stimulation and a dysregulated host immune response, which not only sustains local infection but also damages alveolar and vascular endothelial cells. These pathological changes increase alveolar-capillary permeability, leading to pulmonary edema and, in some cases, lung consolidation—collectively referred to as severe pneumonia-associated lung injury. This condition is a principal cause of hypoxemia and respiratory failure and, in severe cases, may rapidly progress to acute respiratory distress syndrome (ARDS) (1). Global epidemiological data indicate persistently high morbidity and mortality rates, which are influenced by disease severity, patient age, comorbidities, and baseline health status (2–4). Immune mechanisms play a pivotal role in the onset and progression of severe pneumonia. Pathogen invasion triggers immune imbalance, initiating inflammatory responses aimed at pathogen clearance; however, persistent or excessive inflammation aggravates pulmonary injury (5). While current therapeutic strategies—including antimicrobial therapy, respiratory support, and organ function maintenance—have improved survival, interventions specifically targeting immune dysregulation remain inadequate and warrant further exploration.

In this review, we integrate current evidence on infection-immunity mechanisms, molecular regulatory networks, and diagnostic strategies in severe pneumonia-associated lung injury. We discuss key controversies and ongoing challenges, including limitations in translational animal models, the specificity of candidate biomarkers, and pronounced interindividual variability. Finally, we outline future research priorities, emphasizing precision diagnostics, the development of novel disease models, and the incorporation of artificial intelligence(AI)-assisted network pharmacology to accelerate the path toward individualized therapy.

## 2 Infectious immune features of severe pneumonia-associated lung injury

### 2.1 Cytokine storm

The CS in severe pneumonia-related lung injury is not a uniform immune response, but rather a heterogeneous syndrome composed of different immune types. Its core feature is the immune imbalance triggered by infection: under pathogen stimulation, immune cells (such as macrophages and neutrophils) are excessively activated, releasing large amounts of pro-inflammatory factors such as Tumor Necrosis Factor-α (TNF-α), Interleukin-6 (IL-6), and Interleukin-1 beta (IL-1β), leading to a cascading amplification effect (6–8). This dysregulated inflammatory milieu directly injures alveolar and vascular endothelial cells, compromising the integrity of the alveolar-capillary barrier. The resultant increase in permeability promotes pulmonary edema, lung consolidation, hypoxemia, and, in severe cases, systemic multi-organ dysfunction. CS plays a pivotal role in the transition to severe pneumonia-associated lung injury, significantly increasing morbidity and mortality.

Based on studies of lung injury associated with severe pneumonia, several distinct immune endotypes with significant differences can be identified. Each endotype has clear implications for core characteristics, biomarkers, clinical outcomes, and therapeutic strategies, thereby providing a basis for clinically stratified interventions. Details are as follows: The first endotype is the hyperinflammatory endotype. The core mechanism of this endotype is an excessive immune response to infection, tissue damage, or other stimuli: innate immune cells become hyperactivated, triggering disproportionate release of proinflammatory cytokines such as IL-6 and Interleukin-8, initiating an inflammatory cascade that can culminate in multiple organ dysfunction. This picture is accompanied by elevations in C-reactive protein, ferritin, and lactate, and by increased mortality; responses to corticosteroids and to IL-6 blockade vary (9). Treatment for this endotype requires attention to a key time window: early identification of an inflammatory state using biomarkers such as IL-6 and C-reactive protein, and administration within the first 72 hours of illness of an IL-6 receptor antagonist (e.g., tocilizumab) combined with corticosteroids to suppress the inflammatory cascade. IL-6 levels should be monitored dynamically to guide therapy adjustments (9–11). The second endotype is the Neutrophil Extracellular Trap-high endotype. The core pathologic mechanism here is excessive neutrophil activation with release of neutrophil extracellular traps (NETs). NETs contribute to pulmonary microvascular obstruction, release inflammatory proteases and proinflammatory cytokines that directly injure alveolar epithelial and endothelial cells, and activate coagulation pathways that worsen vascular occlusion—ultimately producing pulmonary vascular obstruction and tissue damage (12, 13). Specific NET markers in plasma—such as cell-free DNA, myeloperoxidase–DNA complexes (MPO-DNA), and citrullinated histone H3 (Cit-H3)—are elevated and correlate with disease severity, and therefore may serve as potential stratification biomarkers for NET-targeted interventions (14, 15). The therapeutic time window for this endotype is shorter: within 48 hours of onset, use of NET-degrading agents such as Deoxyribonuclease I together with antiplatelet therapy is recommended to reduce microthrombus formation and mitigate lung injury (16). The third endotype is the immunosuppressive endotype. The hallmark of this endotype is T-cell exhaustion and a predominance of anti-inflammatory mediators, with a markedly increased risk of secondary infections. Core biomarkers include peripheral lymphopenia, upregulation of programmed death-1 (PD-1)/programmed death ligand-1 (PD-L1), elevated Interleukin-10 (IL-10), persistent downregulation of monocyte Human Leukocyte Antigen-DR (HLA-DR), and reduced TNF-α levels (17, 18). This endotype is closely associated with increased ICU-acquired infections and higher long-term mortality (19, 20). Therapeutically, potent anti-inflammatory interventions should be avoided; after effective control of the primary infection, immune-reconstituting strategies—such as administration of Interleukin-7 to restore T-cell function—may be considered around one week after onset to reduce the risk of secondary infection (21). Therefore, a precision mapping of “endotype — therapeutic time window — intervention” should be established. By defining immune endotypes clearly, selecting core stratification biomarkers, and prescribing targeted intervention strategies, clinicians can implement individualized, dynamic therapies for patients with severe pneumonia-associated lung injury, overcoming the limitations of a “one-size-fits-all” approach and providing evidence-based guidance for clinical practice.

### 2.2 Polarization of multiple immune cells

#### 2.2.1 Macrophage polarization

Macrophages are phagocytic cells of the innate immune system that can recognize, engulf, and eliminate foreign pathogens (such as bacteria, viruses, etc.). They also play a crucial role in clearing necrotic cells, regulating immune responses, and maintaining tissue homeostasis. Depending on the activation state in different immune environments, macrophages can differentiate into two main subtypes: M1 type (classically activated) and M2 type (alternatively activated). Both of these macrophage subtypes are associated with various inflammatory diseases (22–24). In severe pneumonia, alveolar macrophages (AMs) and circulating monocytes are predominantly skewed toward the pro-inflammatory M1 phenotype, which exacerbates lung inflammation through the secretion of cytokines such as IL-1β and TNF-α, thereby perpetuating tissue damage (25–29). Zhang et al. (30) utilized multi-omics sequencing technology to screen and identify a novel endogenous double-stranded RNA, named Interferon-β-inducible protein 1 (IBR1), which binds to interferon-induced helicase C domain-containing protein 1 (IFIH1) in lipopolysaccharide (LPS)-induced macrophages. They confirmed that LPS significantly upregulates IBR1 expression, and IBR1, through its double-stranded structure, interacts with the helicase domain of IFIH1 to activate its function. Further experiments showed that the IFIH1-IBR1 signaling axis, with IBR1 as the core initiating molecule, promotes M1 macrophage polarization by activating downstream signals, thereby exacerbating pulmonary inflammatory injury.

#### 2.2.2 Neutrophil polarization

Neutrophil polarization refers to the directional alteration of their functions and phenotypes under inflammatory conditions, whereby their activity shifts from normal anti-infective functions to an excessively activated state, releasing large amounts of cytotoxic substances such as NETs. Over the past decades, NETs have been extensively studied, particularly for their critical role in SARS-CoV-2 infection, and NET levels have been shown to correlate with disease severity (16). NETs exert dual effects: on the one hand, they can trap and facilitate the killing of bacteria, fungi, viruses, and parasites, thereby preventing pathogen dissemination. On the other hand, excessive NETs release amplifies inflammation and induces tissue injury (16). Zheng et al. (31) reported in a severe pneumonia model using SARS-CoV-2-infected human Angiotensin-converting enzyme 2 transgenic mice that neutrophils are massively recruited to the lungs under the influence of chemokines and polarize toward a pro-inflammatory phenotype, forming functionally heterogeneous subpopulations such as Cd177^high^ and Stfa^high^, which exhibit high expression of pro-inflammatory genes including IL-1β and TNF. Additionally, NETs detected in both murine and human lung tissues were found to exacerbate lung injury and suppress adaptive immunity by depleting arginine, highlighting the pivotal role of neutrophils in SARS-CoV-2-induced severe pneumonia. Collectively, these findings indicate that NETs are a key contributor to the exacerbation of lung injury in severe pneumonia, and modulation of this process may provide a potential therapeutic target.

### 2.3 Role of pathogen-associated molecular patterns, damage-associated molecular patterns, and aconitate decarboxylase 1 in immune inflammation

PAMPs and DAMPs are critical initiators of innate immune responses. PAMPs are recognized by pattern recognition receptors, providing the first line of defense against invading pathogens, whereas DAMPs, released during tissue injury, activate innate immune pathways and may disrupt immune homeostasis (32). Karki et al. (33) demonstrated that both PAMPs and DAMPs can induce cellular pyroptosis through a positive feedback mechanism, thereby creating a self-perpetuating cycle of inflammatory cell death and cytokine release that may ultimately lead to multi-organ failure. Beyond the classical sequence in which PAMPs and DAMPs recognition leads to immune activation and subsequent tissue injury, the Toll-like Receptor (TLR)/Nuclear Factor-κB (NF-κB) signaling axis also plays an important role in the inflammatory response.

ACOD1 is an innate immune gene regulated by TLR, Interferon-α Receptor, and NF-κB signaling pathways. ACOD1 expression is upregulated in activated immune cells in response to pathogens, PAMPs, cytokines, and DAMPs. Wu et al. (34) focused on the immune metabolic gene ACOD1, highlighting its emerging role as a regulator in immune metabolism during inflammation and infection, and revealing its “double-edged sword” effect in infections, inflammation, and cancer. ACOD1 encodes aconitate decarboxylase, an enzyme that catalyzes the production of itaconate—a metabolite with context-dependent immunomodulatory effects. ACOD1 participates in the immune response by regulating itaconate metabolism and oxidative stress, with its action highly dependent on the microenvironment. Itaconate can activate the Nuclear factor erythroid 2-related factor 2 pathway by alkylating Kelch-like ECH-associated protein 1 or regulate downstream TLRs/NF-κB signaling to suppress inflammation (35, 36). Itaconate exerts a bidirectional effect on TLR/NF-κB-mediated inflammation regulation: on one hand, under conditions such as LPS stimulation, itaconate reduces the release of pro-inflammatory factors like IL-1β and TNF by inhibiting the activity of succinate dehydrogenase, thus alleviating excessive inflammatory damage (37–39). On the other hand, in models of Leishmania or vesicular stomatitis virus infections, itaconate may enhance pathogen survival and replication by metabolic reprogramming, promoting protein isoprenylation, or excessively inhibiting the NF-κB pathway, thereby weakening host defense (40–42). Wang et al. (43) using a mouse model of Mycoplasma pneumoniae pneumonia, clinical samples, *in vitro* neutrophil assays, and targeted intervention experiments, demonstrated that Mycoplasma pneumoniae infection significantly upregulated ACOD1 (IRG1) expression in lung tissue and itaconate levels in bronchoalveolar lavage fluid, which were positively correlated with the severity of lung inflammation. Neutrophils were the main source of itaconate under infection, and Mycoplasma pneumoniae induced high expression of IRG1 in neutrophils through the TLR2/MyD88/NF−κB/STAT1 signaling pathway. Mechanistically, itaconate decreased mitochondrial reactive oxygen species (mtROS) production by inhibiting succinate dehydrogenase activity, thereby weakening the bactericidal ability of neutrophils and prolonging neutrophil survival by blocking mtROS−dependent apoptosis, which aggravated lung inflammation and tissue damage. Moreover, IRG1 knockout, IRG1 inhibitors, or mtROS activation significantly attenuated lung inflammation and reduced bacterial burden. This bidirectional effect has significant translational medical implications: from a temporal perspective, moderate itaconate production in the early stages of infection can prevent an inflammatory storm, whereas persistent high expression in the later stages may hinder pathogen clearance, necessitating precise intervention timing. From a dosage perspective, physiological levels of itaconate maintain a balance between inflammation and immune defense, while excessive levels may induce immune suppression. In clinical applications, when itaconate derivatives or ACOD1 modulators are used in combination with antibiotics, the treatment regimen should be adjusted based on the type of infectious pathogen, with individualized regulation to achieve therapeutic benefit (44–47).

## 3 Hierarchical resolution of molecular regulatory networks

### 3.1 Activation and regulation of signaling pathways

**Figure 1:**
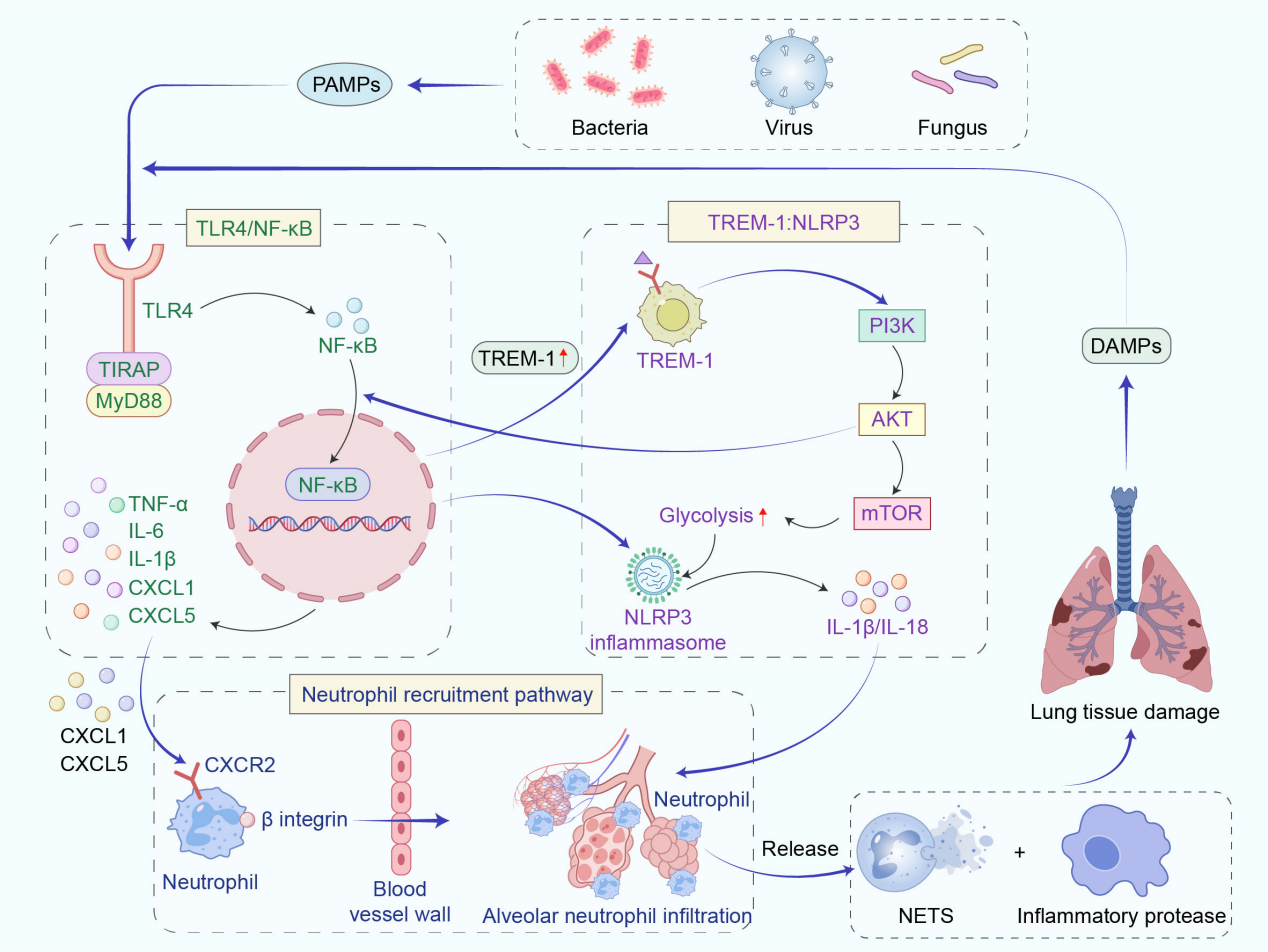
Cascade regulatory mechanism of infection-mediated pulmonary inflammation. This figure depicts the cascade of infection-induced pulmonary inflammation amplification by pathogens (bacteria, viruses, fungi): Pathogen PAMPs bind TLR4, activating NF-κB via TIRAP/MyD88 to drive proinflammatory factors (TNF-α, IL-1β) and chemokines (CXCL1/5). Upregulated TREM-1 enhances glycolysis via PI3K/AKT/mTOR, activating the NLRP3 inflammasome to release IL-1β/IL-18 and amplify inflammation. CXCL1/5 recruits neutrophils via CXCR2; neutrophils infiltrate alveoli via β-integrins, releasing NETs and proteases to induce lung injury. DAMPs from damaged tissue further aggravate inflammation, forming a positive feedback loop of "pathogen recognition-signal amplification-cell infiltration-tissue injury.".

The pathological process of severe pneumonia-related lung injury is regulated by three signaling pathways: the pattern recognition receptor-related pathway, which recognizes pathogens or damage signals to initiate the inflammatory cascade; the chemokine-mediated migration pathway, which drives the recruitment of inflammatory cells to lung tissue, amplifying local injury (see **Figure 1**); and the resolution of inflammation and immune regulation pathways, which regulate inflammation resolution through specific pro-resolution mediators or immune cells, maintaining inflammatory homeostasis and promoting lung tissue repair.

#### 3.1.1 Pattern recognition receptor-related pathways

The NOD-like Receptor Pyrin Domain Containing 3 (NLRP3) inflammasome is a critical component of the innate immune system and has been implicated in a wide range of inflammatory disorders, with particular significance in severe pneumonia-associated lung injury. In AMs, excessive NLRP3 activation drives an uncontrolled inflammatory response (48, 49). Gupta et al. (50) demonstrated that mitochondrial autophagy and the ubiquitin-proteasome system serve as negative regulators of NLRP3 activity, whereas mitochondrial damage provides a potent activation signal. Zhong et al. (51) found that triggering receptor expressed on myeloid cells 1 promotes NLRP3 activation by enhancing glycolytic flux through the phosphoinositide 3-kinase/protein kinase B/mechanistic target of rapamycin/hypoxia-inducible factor-1α pathway. Once activated, the NLRP3 inflammasome mediates the maturation and secretion of IL-1β and Interleukin-18 (IL-18), amplifying pulmonary inflammation and tissue damage. Moderate activation of the NLRP3 inflammasome can maintain epithelial integrity and support mucosal repair. For example, Fang et al (52). found in a mouse model of pneumococcal infection that NLRP3 promotes the expression of Trefoil Factor 2 and Intelectin-1 through the Signal transducer and activator of transcription 6–SAM pointed domain containing ETS transcription factor axis, thereby facilitating epithelial healing and mucosal immunity, enhancing the host’s ability to combat infection. Therefore, when considering NLRP3 inhibition therapy, monitoring IL-1β activity and signals of secondary infections is crucial to balance therapeutic benefits and potential risks.

Similarly, the TLR4/NF-κB signaling axis plays a central role in the immune response to Gram-negative bacterial infections. LPS binds to the Toll-like receptor 4–myeloid differentiation factor 2 complex and activates Inhibitor of nuclear factor kappa-B kinase through the Myeloid differentiation primary response gene 88-dependent pathway, leading to Inhibitor of nuclear factor kappa-B alpha degradation and subsequent nuclear translocation of NF-κB. This process induces the transcriptional upregulation of pro-inflammatory cytokines such as TNF-α and IL-6, as well as chemokines like C-X-C motif chemokine ligand 1 (CXCL1) and C-X-C motif chemokine ligand 5 (CXCL5), thereby driving neutrophil infiltration and the inflammatory response (53). More importantly, the TLR4/NF-κB signaling axis and NLRP3 inflammasome exhibit a tight synergistic interaction: TLR4/NF-κB can lay the foundation for NLRP3 activation through transcriptional regulation, while the IL-1β and IL-18 produced by NLRP3 activation can enhance the TLR4/NF-κB signaling via positive feedback (54). Synergistic crosstalk between TLR4/NF-κB signaling and NLRP3 inflammasome activation shapes both the magnitude and duration of the inflammatory response, underscoring the interdependence of these innate immune pathways in the pathophysiology of severe pneumonia-associated lung injury.

#### 3.1.2 Chemokine-mediated migration pathway

Chemokines orchestrate neutrophil recruitment primarily via the CXCL1/CXCL5-C-X-C motif chemokine ligand 2 (CXCL2) axis and β1-integrin-mediated adhesion. In response to pathogen recognition, alveolar epithelial cells secrete CXCL1 and CXCL5, which engage CXCR2 on neutrophils to drive chemotaxis into the alveolar spaces. Herbold et al. (55) conducted experiments using CXCR2 knockout mice, 10%-75% CXCR2-deficient bone marrow chimeric mice, and the CXCR2 antagonist SB-225002. They found that impaired CXCR2 function inhibits the recruitment of neutrophils and macrophages to the lungs, while triggering a chemokine storm involving CXCL1, CXCL2, and other chemokines. This leads to insufficient phagocyte numbers and dysfunction in the lungs, resulting in reduced bacterial clearance, uncontrolled pathogen proliferation in lung tissue, and a significant increase in infection-related mortality. These findings suggest that the CXCR2 axis plays an important role in maintaining lung defense and prognosis after pneumococcal infection. During Streptococcus pneumoniae infection, β1-integrins on neutrophils mediate firm adhesion to the vascular endothelium, facilitating transmigration into lung tissue. Overactivation of both the CXCL1/CXCL5-CXCR2 axis and β1-integrin-dependent adhesion can amplify inflammatory responses and worsen tissue injury. Kadioglu et al (56)., using a gene knockout mouse model of pneumococcal infection and antibody blockade experiments, found that β1 integrin alone mediates T cell recruitment to the lungs and collaborates with macrophage-1 antigen to facilitate neutrophil migration. Its ligand, vascular cell adhesion molecule 1, is initially localized to the bronchial epithelium during early infection and later expands to the vascular walls, providing targeted migration sites for immune cells. Disrupting this pathway results in a significant increase in bacterial load in both lung tissue and blood, exacerbating the spread of infection. Therefore, the β1 integrin pathway plays a crucial role in regulating the directional transport of immune cells and maintaining lung defense, making it an important protective pathway in pneumococcal infections.

#### 3.1.3 Inflammatory resolution and immunoregulatory pathways

**Figure 2:**
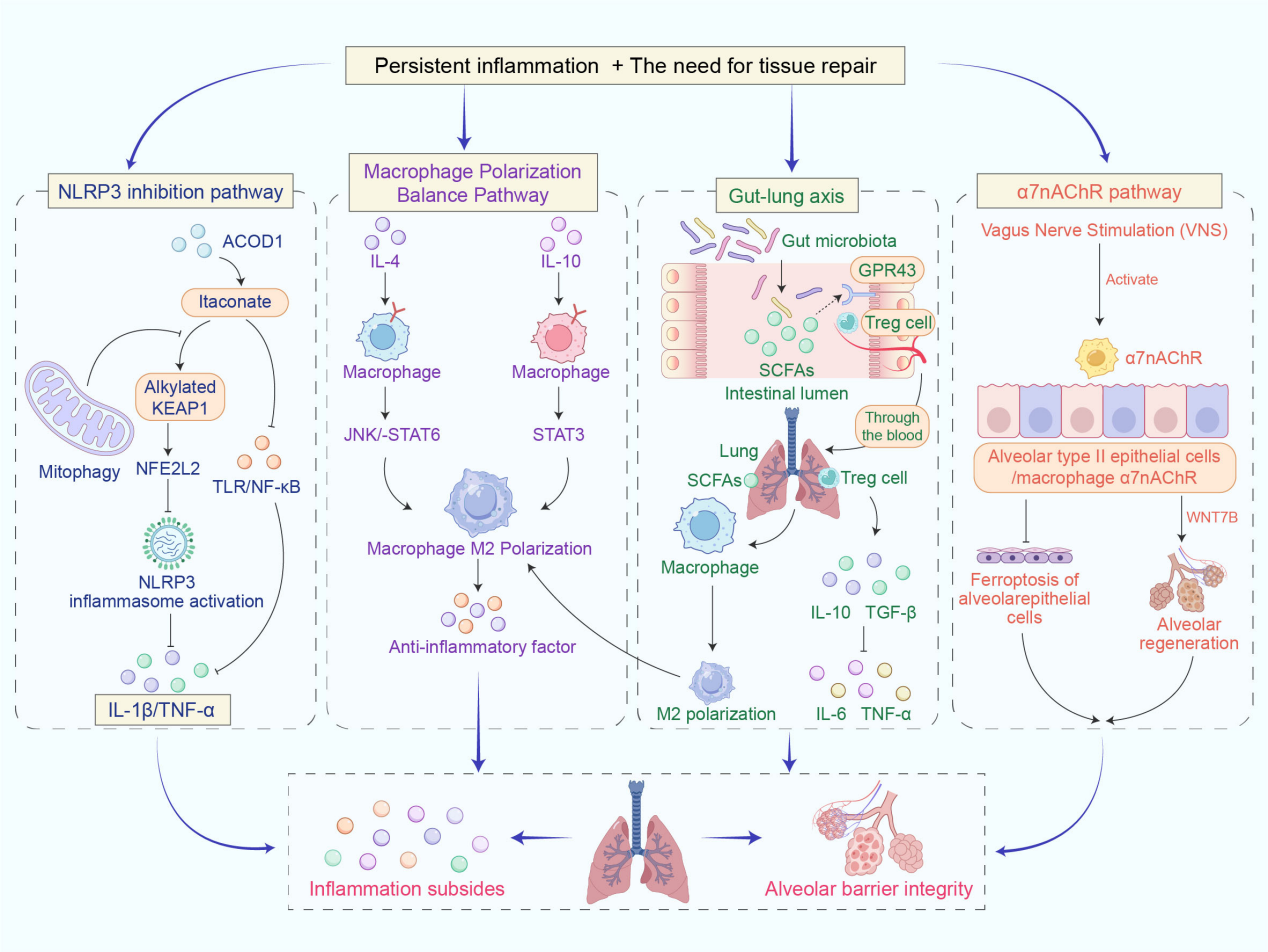
Multi-pathway regulatory mechanisms mediating inflammation resolution and tissue repair in pulmonary injury. This figure illustrates four pathways—the inhibition of NLRP3, macrophage M2 polarization, the gut-lung axis, and the α7nAChR pathway—that synergistically suppress pulmonary inflammation, promote the anti-inflammatory phenotype of macrophages, and facilitate alveolar regeneration, ultimately achieving inflammation resolution and the restoration of alveolar barrier homeostasis.

Resolution of inflammation is governed by the coordinated activity of multiple immunomodulatory pathways (see **Figure 2**). Kumar et al. (32) reported that, in severe pneumonia-associated lung injury, AMs and TLR signaling promote polarization toward the M2 phenotype through pathways such as IL-10/Signal Transducer and Activator of Transcription 3. This shift is driven in part by neutrophil infiltration, which facilitates the release of anti-inflammatory mediators and clearance of apoptotic cells. However, this resolution phase may carry the risk of excessive immunosuppression. Ouyang et al. (57) used myeloid cell-specific Shp2 conditional knockout mice to establish a post-influenza Staphylococcus aureus pneumonia model. They found that, in knockout mice, lung macrophages significantly polarized to the M2 phenotype, with elevated levels of type I interferon and reduced secretion of chemokines keratinocyte-derived chemokine and macrophage inflammatory protein 2. This resulted in insufficient neutrophil infiltration and impaired bacterial clearance, leading to a lower survival rate after secondary infection. Exogenous supplementation of keratinocyte-derived chemokine and macrophage inflammatory protein 2 could restore the relevant functions, indicating that M2 macrophages weaken bacterial clearance by inhibiting neutrophil recruitment, thereby increasing the risk of secondary infections. Mengos et al. (58) further discovered that CD14-positive HLA-DR (CD14+HLA-DR) low-expressing monocytes are key mediators in the conversion of M2 polarization to immune suppression. These monocytes regulate the downregulation of HLA-DR expression via cytokines such as IL-1β, IL-10, and transforming growth factor-beta (TGF-β), facilitating the transformation of monocytes from a pro-inflammatory phenotype to an anti-inflammatory phenotype, thereby mediating the shift of overall inflammatory status toward immune suppression (59–63). Therefore, when intervening to induce M2 macrophage polarization, it is essential to assess the immune status using operable monitoring indicators. Standardized flow cytometry should be used to detect CD14+HLA-DR expression, while monitoring peripheral blood lymphocyte counts to provide early warning of immune suppression and the risk of secondary infections.

AMs exhibit dynamic functional transitions during inflammation: initially releasing pro-inflammatory mediators such as TNF-α and reactive oxygen species to initiate the immune response, and subsequently suppressing proinflammatory signals while promoting anti-inflammatory mediators, including lipoxin A4 and growth arrest-specific 6, to facilitate resolution (64). In addition, innate lymphoid cells contribute to immunoregulation through subset-specific mechanisms. type 1 innate lymphoid cell and natural killer cells enhance macrophage bactericidal activity via interferon-γ secretion, whereas type 3 innate lymphoid cell mitigate inflammatory responses and promote pathogen clearance through the production of Interleukin-22 and Interleukin-17 (65). These observations highlight the complexity of immune regulation in pneumonia-associated lung injury.

### 3.2 Epigenetic regulation

The epigenetic regulation of severe pneumonia-related lung injury refers to the modulation of immune cell functions, inflammatory factor expression, and pulmonary tissue homeostasis through mechanisms such as DNA methylation, histone modifications, and non-coding RNAs. Observational studies and perturbation experiments have both demonstrated that these mechanisms are closely associated with disease progression and play a role in the onset, development, and repair of pulmonary inflammation.

#### 3.2.1 DNA methylation regulation

DNA methylation is one of the key mechanisms in epigenetic regulation. Hypomethylation of promoter regions in inflammation-related genes (e.g., IL-6, TNF-α) is associated with increased transcriptional activity and elevated production of pro-inflammatory mediators, thereby aggravating pulmonary inflammation (66). Aberrant methylation of genes encoding barrier-protective proteins, including tight junction components in lung epithelial cells, can compromise the alveolar-capillary barrier and exacerbate lung injury. In an observational study, Morselli et al. (67) used targeted DNA methylation approaches to analyze peripheral blood from pneumonia patients (including both COVID-19 positive and negative cases). They found that changes in DNA methylation regions were associated with variability in immune cell composition and the severity of pneumonia, suggesting their potential application in disease prediction.

#### 3.2.2 Histone modification regulation

Histone modifications play a crucial regulatory role in lung inflammation by modulating transcriptional activity and shaping immune cell function. Elevated histone acetylation at the promoters of pro-inflammatory genes, such as NF-κB targets, enhances transcriptional activity, thereby promoting neutrophil infiltration and cytokine release that exacerbate pulmonary injury (68). In contrast, aberrant histone deacetylase (HDAC) activity may reduce acetylation at anti-inflammatory loci, including the IL-10 promoter, leading to impaired transcription and hindering the resolution of inflammation (66). Moreover, histone methylation modifications regulate immune cell functions by methylating specific lysine residues (such as histone H3 lysine 4 trimethylation activating genes and histone H3 lysine 27 trimethylation repressing genes), which mediate immune cell polarization (e.g., macrophage M1/M2 phenotype switching). Ultimately, these modifications regulate the onset and resolution of pulmonary inflammation (69). Rolando et al. (70) conducted perturbation experiments with Legionella effector RomA and Legionella histone deacetylase proteins, along with ChIP-seq analysis, and found that effector proteins secreted by Legionella pneumophila can modify histone H3 lysine 14. Legionella effector RomA protein mediates histone H3 lysine 14 methylation, while Legionella histone deacetylase protein mediates histone H3 lysine 14 deacetylation. Together, these modifications suppress the expression of immune response genes, aiding bacterial evasion. This study clarified the regulatory role of histone modifications in severe pneumonia-related lung injury.

#### 3.2.3 Non-coding RNA regulation

Non-coding RNA, including microRNA (miRNA) and long non-coding RNA, orchestrate post-transcriptional regulation of immune and inflammatory processes (71). For example, microRNA-155 (miR-155) promotes pro-inflammatory responses by targeting negative regulators of inflammation (72). Tian et al. (73), through clinical sample analysis combined with animal model experiments, found that in methicillin-resistant Staphylococcus aureus pneumonia, miR-155 promotes Th17 cell differentiation and Interleukin-17 secretion by targeting and suppressing the transcription factor forkhead box protein P3, thereby exacerbating pulmonary inflammation. In hypervirulent Klebsiella pneumoniae-induced acute lung injury, Xu et al. (74) established a mouse model by intravenously injecting inactivated hypervirulent Klebsiella pneumoniae and discovered that exosomal microRNA-155-5p exacerbates inflammation by promoting macrophage M1 polarization via the Mitogen-Activated and Stress-Activated Kinase 1/p38 Mitogen-Activated Protein Kinase pathway. MicroRNA-21 has been reported to inhibit inflammation through modulation of the TLR4/NF-κB axis, although its dysregulation may contribute to lung injury progression in certain contexts. Long non-coding RNA also influence disease outcomes by regulating chemokine expression in neutrophils and modulating macrophage function, often through competing endogenous RNA networks or direct interactions with DNA and proteins.

Collectively, these epigenetic mechanisms dynamically regulate gene expression, influencing the intensity of immune responses, the extent of lung tissue damage, and the repair capacity in severe pneumonia-related lung injury, thus offering new potential directions for targeted therapy.

### 3.3 Cross-organ interaction networks

#### 3.3.1 Gut-lung axis regulatory mechanisms

The gut microbiota exerts profound effects on respiratory immunity through the bidirectional gut-lung axis. Dysbiosis of intestinal microbiota is strongly linked to the onset and exacerbation of respiratory diseases, as demonstrated in both human and animal models (75, 76). Microbial communities and their metabolites—particularly short-chain fatty acids (SCFAs)—modulate lung immunity through multiple mechanisms. SCFAs can enter systemic circulation to influence alveolar macrophage function, while gut-associated lymphoid tissue-activated immune cells migrate to the lung to exert immune-modulatory effects. For instance, regulatory T cells (Tregs) induced by gut microbiota metabolites (such as SCFAs) can migrate from the gut to the lungs via the bloodstream, modulating the anti-inflammatory M2 polarization of alveolar macrophages, thereby maintaining immune homeostasis and protecting the alveolar barrier (77–79). Respiratory viral infections may disrupt gut microbiota composition and reduce SCFAs production, increasing susceptibility to secondary infections (80, 81). There are conflicting results regarding the anti-respiratory viral effects of SCFAs. On one hand, mouse experiments have shown that gut microbiota-derived SCFAs may inhibit viral replication by modulating type I interferons (interferon-alpha/beta) and cluster of differentiation 8-positive T cell metabolism (82). On the other hand, *in vitro* experiments have indicated that SCFAs do not directly inhibit infection in intestinal epithelial cells (83), suggesting that the effects of SCFAs in viral infections may vary depending on factors such as the site of infection and the type of virus. Additionally, mouse experiments have confirmed that a high-fiber diet increases the production of SCFAs (acetate, propionate, butyrate), and through the activation of G protein-coupled receptor 43, it enhances the differentiation of regulatory T cells. These Tregs migrate to the lungs, secreting anti-inflammatory factors such as IL-10 and TGF-β, while inhibiting the release of pro-inflammatory factors like IL-6 and TGF-α. Consequently, this process alleviates pulmonary inflammation and maintains the integrity of the alveolar barrier (84, 85).

Tang et al. (86) established a mouse model in which fecal microbiota transplantation has shown potential in improving outcomes in Klebsiella pneumoniae pneumonia, including enhanced survival, reduced inflammation, preserved epithelial barrier integrity, decreased antibiotic resistance, and restoration of beneficial metabolites such as SCFAs. Sencio et al. (87) further demonstrated, through a combination of mouse model experiments and a small human cohort study, that respiratory viral infections, including COVID-19, can induce intestinal dysbiosis, exacerbating disease severity and increasing the risk of secondary bacterial infections. These findings support the therapeutic potential of strategies targeting the gut-lung axis for both bacterial and viral pneumonia.

#### 3.3.2 Neuro-immune axis: Vagal-α7 nicotinic acetylcholine receptor anti-inflammatory pathway

α7nAChR is a key molecular mediator of neuroimmune regulation with protective effects in inflammatory diseases affecting multiple organs. In respiratory diseases, α7nAChR, as the core molecule of the cholinergic anti-inflammatory pathway, plays a key role in regulating immune cell function, controlling the inflammatory response, and coordinating immune responses across different organs via cholinergic anti-inflammatory pathway. It shows potential for the treatment of various inflammatory diseases, but its effects on specific immune cell subpopulations still require further investigation (88). Zhang et al. (89) found through *in vivo* and *in vitro* experiments in mice that electroacupuncture specifically activates α7nAChR, inhibiting alveolar epithelial cell ferroptosis through the regulation of key ferroptosis pathways, thereby alleviating LPS-induced ARDS. Yu et al. (90) confirmed in mouse experiments that the traditional Chinese medicine compound Liangge San upregulates α7nAChR expression and alleviates virus-induced acute lung injury (ALI) by inhibiting mitochondrial autophagy. Chen et al. (91) further revealed through *in vivo* and *in vitro* experiments in mice that α7nAChR in type II alveolar cells (AT2) promotes alveolar regeneration via the Wnt family member 7B signaling pathway, providing a new direction for lung injury repair. It should be noted that there is currently limited research evidence for α7nAChR in humanized ALI, and its mediating effect on lung regeneration has not been specifically validated in clinical studies (92). Animal experiments have shown that the activation of α7nAChR is most effective during the repair phase following injury (such as the proliferation and differentiation of AT2 cells) (91). Based on this finding, clinical studies should prioritize recruiting early-stage patients with AT2 dysfunction within 72 hours of onset, or patients in the recovery phase with ongoing alveolar repair defects after inflammation has been controlled (93, 94). The patient’s recovery can be structurally assessed using high-resolution computed tomography and functionally assessed with diffusing capacity of the lung for carbon monoxide. Additionally, AT2 cell proliferation markers such as Ki67 antigen can also be monitored (91, 94, 95). The research strategy suggests initially conducting Phase I safety trials, followed by Phase II concept verification in patients with AT2 dysfunction.

In conclusion, α7nAChR may become a potential therapeutic target for severe pneumonia-associated lung injury through a “multi-mechanism-multi-organ” synergistic mode, laying an important foundation for developing personalized anti-inflammatory therapies targeting α7nAChR. However, clinical application must first address issues of humanized mechanism validation and long-term safety, and future trials should focus on populations with AT2 cell dysfunction to clarify its therapeutic value in humanized ALI.

## 4 Intervention strategies for targeted regulatory networks

To systematically present the mechanisms, research progress, and limitations of drug therapies targeting precise timing interventions and key nodes in severe pneumonia-related lung injury (see **Table 1**).

**Table 1:** Targeted drugs for severe pneumonia-associated lung injury.

Research team	Experimental model	Representative drug	Intervention strategy / target	Mechanism of action	Clinical translation stage	Limitations and considerations
[Cheng et al.] (96)	Angiotensin-Converting Enzyme 2-expressing Rat Lung Epithelial-6-T-antigen Negative cell model	BAY 11−7082	Block NF-κB pathway	By inhibiting IκBα phosphorylation and degradation, reducing p65 phosphorylation and nuclear translocation, it suppresses NF-κB-mediated release of pro-inflammatory cytokines such as TNF-α and IL-6, thereby alleviating pulmonary inflammatory damage	Preclinical research stage	Long-term use of BAY 11-7082 to suppress NF-κB may increase the risk of infection or impaired tissue repair
[Lv et al.] (97)	A mouse model of pulmonary fibrosis induced by bleomycin	Pirfenidone	Inhibition of TGF-β/Smad pathway	By inhibiting the TGF-β/Smad pathway, the synthesis of pro-inflammatory factors such as TGF-β, TNF-α, and IL-6 is reduced, thereby decreasing fibroblast proliferation and collagen synthesis, which alleviates fibrosis	Post listing monitoring	Common side effects include gastrointestinal discomfort, photosensitive rash and elevated liver enzyme levels, requiring regular monitoring
[Tapia-Abellán et al.] (98)	Mouse macrophage	MCC950	Inhibition of NLRP3 inflammatory bodies	By targeting the Natch domain of NLRP3, this mechanism prevents its conformational changes and oligomerization, thereby inhibiting inflammatory microbody activity, reducing pro-inflammatory factor release, and decreasing inflammatory infiltration in lung tissue	Preclinical research stage	Serum liver enzyme elevation induced by ultra-high doses, and reduced IL-1β release from NLRP3 inflammasome inhibition leading to decreased susceptibility to infection
[Kasotakis et al.] (99)	The mouse model of acute lung injury induced by pneumonia of Gram-negative bacteria	Trichostatin A	Inhibition of HDAC	By inhibiting HDAC7 activity, remodeling chromatin structure, and downregulating pro-inflammatory gene expression: improving inflammatory phenotype and enhancing survival rate	Preclinical research stage	The lack of absolute specificity prevents exclusion of cross-effects on multiple HDAC subtypes; the anti-inflammatory effect is limited to specific inflammatory factors and is uneven
[Fu et al.] (100)	Cecal Ligation and Puncture-induced septic ALI mouse model	Anti-miR-155	miRNA antagonist	By specifically binding to miR-155, it blocks the interaction between miR-155 and target mRNAs, thereby inhibiting excessive macrophage activation, reducing the release of pro-inflammatory factors such as IL-6 and MIP-2 from alveolar macrophages, upregulating the expression of the anti-inflammatory factor IL-10, and alleviating pulmonary inflammatory injury through suppressing inflammatory factor secretion	Preclinical research stage	It has off-target effects and immune stimulation; needs optimization of lung-targeted delivery carriers (e.g., lipid/nanocarriers) and evaluation of pathogen clearance risks

### 4.1 Timing and precision intervention

The pathological progression of severe pneumonia-related lung injury is characterized by “cascading amplification and stage-specific differentiation,” and sequential interventions must align with the core driving mechanisms at each stage (101–103). Interventions should be phased precisely, based on drug evidence levels, core risks, and biomarker anchoring windows.

#### 4.1.1 Early stage (acute inflammatory surge): targeting the NF-κB pathway to block the inflammatory storm

As a key transcription factor regulating the inflammatory response, NF-κB has been studied by Cheng et al. (96) using an Angiotensin-Converting Enzyme 2-expressing Rat Lung Epithelial-6-T-antigen Negative cell model. They found that BAY 11–7082 inhibits the phosphorylation and degradation of Inhibitor of nuclear factor kappa-B alpha, reduces p65 phosphorylation, and prevents nuclear translocation, thereby suppressing NF-κB-mediated release of pro-inflammatory cytokines such as TNF-α and IL-6, alleviating lung tissue inflammation. However, NF-κB is widely involved in immune homeostasis, and long-term inhibition of NF-κB with BAY 11–7082 may increase the risk of infections or impair tissue repair. Therefore, clinical use requires strict monitoring of early inflammatory markers such as IL-6 and TNF-α to anchor the intervention window, while also being cautious of the risk of immune suppression (104–106).

#### 4.1.2 Middle stage (critical immune remodeling period): regulating macrophage polarization balance

Macrophages maintain a dynamic balance between M1 (pro-inflammatory) and M2 (anti-inflammatory repair) polarization. Overactivation of M1 exacerbates lung damage. During this stage, macrophages can be polarized from M1 to M2 to mitigate lung injury through induction by Interleukin-4, among other methods. Numerous studies using mouse models have shown that Interleukin-4 drives M2 polarization through Signal transducer and activator of transcription 6 phosphorylation, thereby protecting lung tissue (107, 108). However, excessive Interleukin-4 levels may lead to tissue fibrosis (109). Thus, during clinical application, dynamic monitoring of peripheral blood p-Signal transducer and activator of transcription 6 and other markers is necessary to avoid excessive M2 polarization, which may induce lung tissue fibrosis (107, 110).

#### 4.1.3 Late stage (tissue repair and reconstruction period): promoting alveolar repair and inhibiting fibrosis

In the late stage, the core focus is on promoting alveolar repair and inhibiting fibrosis. During this period, the activation of fibroblast growth factor and hepatocyte growth factor pathways can promote the proliferation and differentiation of type II alveolar epithelial cells (111, 112). For individuals with a tendency toward fibrosis, pirfenidone can be used to inhibit the TGF-β/Smad pathway, thereby suppressing the synthesis of TGF-β, TNF-α, IL-6, and other pro-inflammatory cytokines. This reduces fibroblast proliferation and collagen synthesis, alleviating fibrosis (113). Lv et al. (97) established a mouse model of pulmonary fibrosis using bleomycin and also confirmed that pirfenidone can alleviate pulmonary fibrosis both *in vitro* and *in vivo* by regulating the TGF-β/Smad signaling pathway. In clinical practice, pirfenidone is a first-line standard treatment for idiopathic pulmonary fibrosis worldwide, with common side effects including gastrointestinal discomfort, photosensitive rash, and elevated liver enzyme levels (114). When using pirfenidone, TGF-β levels in blood or bronchoalveolar lavage fluid should be closely monitored, and regular indicators such as Forced Vital Capacity, High-Resolution Computed Tomography, and liver function should be assessed to monitor both efficacy and safety (113, 115).

### 4.2 Key node targeting

#### 4.2.1 NLRP3 inflammasome inhibitors

Activation of the NLRP3 inflammasome promotes the maturation and release of pro-inflammatory cytokines, notably IL-1β and IL-18, thereby amplifying inflammatory damage in lung tissue. MCC950 is a compound that specifically inhibits the NLRP3 inflammasome (116). Tapia-Abellán et al. (98) found in a mouse macrophage model that MCC950 targets the NATCH domain of NLRP3, preventing conformational changes and oligomerization of NLRP3, thereby inhibiting NLRP3 inflammasome activity, reducing the release of pro-inflammatory cytokines, and significantly decreasing inflammatory infiltration in lung tissue. The limitations of MCC950 include hepatotoxicity at very high doses (manifested by elevated serum liver enzymes) and increased susceptibility to infection resulting from inhibition of the NLRP3 inflammasome and consequent reduction of IL-1β release. Together, these issues have impeded its clinical translation, leaving it still at the preclinical research stage (117). Future work will require the development of appropriate strategies to address these challenges and enhance its translational potential. Beyond direct inhibition, post-transcriptional regulation of NLRP3 also influences inflammasome activity. Cao et al. (118) established an acute lung injury mouse model through intraperitoneal LPS injection and found that methyltransferase-like protein 14 (METTL14) stabilizes NLRP3 mRNA via an N6-methyladenosine–insulin-like growth factor 2 mRNA-binding protein 2 (IGF2BP2) pathway. METTL14 knockdown reduced NLRP3 expression and downstream cytokine release, attenuating lung injury, whereas METTL14 overexpression exacerbated inflammation. These findings highlight METTL14 and IGF2BP2 as potential upstream targets for modulating NLRP3-driven pathology. The target is currently in preclinical development, with the target’s clinical efficacy, NLRP3’s N6-methyladenosine methylation sites, and the upstream mechanisms of METTL14/IGF2BP2 upregulation requiring further investigation. Overall, NLRP3 inhibition presents a “double-edged sword” effect: while inhibiting NLRP3 in acute inflammation can alleviate inflammation-mediated tissue damage (119), long-term or chronic inhibition may interfere with normal tissue repair processes (120). Therefore, future clinical translation will require dynamic monitoring of inflammation factor levels (such as IL-1β, IL-18), pathogen type, and the extent of lung tissue damage, in order to optimize the timing and dosage of interventions and achieve a balanced regulation of inflammation suppression and host defense.

#### 4.2.2 HDAC inhibitors

HDAC inhibitors modulate chromatin architecture by blocking deacetylase activity, thereby reprogramming the transcriptional landscape that governs pro-inflammatory and anti-inflammatory gene expression and, in the setting of pulmonary injury, downregulating mediators such as TNF-α to attenuate lung inflammation (121). Among these enzymes, the class IIa member histone deacetylase 7 is upregulated in inflammatory macrophages and exerts broad control over cellular metabolism and inflammatory signaling (73). In a murine model of ALI precipitated by Gram-negative pneumonia, Kasotakis et al. (99) administered the pan-HDAC inhibitor trichostatin A and observed a marked mitigation of pulmonary inflammatory pathology accompanied by improved survival, implicating histone deacetylase 7 in the host inflammatory response to severe Gram-negative pneumonia-associated lung injury. While trichostatin A alleviated inflammation and improved survival rates in ALI induced by Gram-negative pneumonia, the study revealed several critical limitations: its lack of absolute specificity prevents the exclusion of cross-effects on multiple HDAC subtypes; the anti-inflammatory effect targets only certain inflammatory factors (e.g., TNF-α, IL-6), with an uneven distribution of effects; and factors such as the need for systemic administration, narrow dosing windows, and potential unidentified toxicity all hinder its clinical translation potential.

#### 4.2.3 miRNA antagonists

MiRNA such as miR-146a/b, miR-155, and miR-132 are critical modulators of inflammatory signaling (122–125). Among these, miR-155 acts as a potent amplifier of inflammation by suppressing negative regulators of immune activation (72). By establishing the Cecal Ligation and Puncture-induced septic ALI mouse model, Fu et al. (100) discovered that in inflammatory diseases such as pneumonia, the upregulation of miR-155 expression promotes the overactivation of immune cells such as macrophages, exacerbating the inflammatory response by promoting the production and release of pro-inflammatory cytokines like TNF-α and IL-6. MiR-155 antagonists (e.g., anti-miR-155) can specifically bind to miR-155, blocking its interaction with target mRNAs, thereby inhibiting macrophage overactivation. This leads to a reduction in the release of pro-inflammatory factors such as IL-6 and macrophage inflammatory protein-2 from AMs, upregulation of the anti-inflammatory factor IL-10, and decreased release of inflammatory cytokines, alleviating the inflammatory cascade. However, miRNA antagonist therapy poses challenges, including off-target effects, immune stimulation, and carrier-related toxicity. To enhance pulmonary targeting and reduce the risk of systemic off-target effects, studies have explored inhalation/aerosol delivery or local administration via the airways, combined with carriers such as lipids, nanoparticles, or DNA tetrahedra to improve lung deposition and stability (126, 127). Therefore, when administering miRNA antagonist therapy for patients with severe pneumonia-related lung injury, careful evaluation of its anti-inflammatory benefits versus the potential risk of impairing the host’s ability to clear pathogens is necessary (128, 129).

In terms of clinical translation, the above-mentioned NLRP3 inhibitors, HDAC inhibitors and miRNA antagonists remain at the preclinical stage, with no registered clinical trial data for severe pneumonia-related lung injury. MCC950, a representative NLRP3 inhibitor, has been discontinued in clinical development due to high-dose hepatotoxicity and infection susceptibility, without relevant studies on pulmonary inflammation or injury (130, 131). Current HDAC inhibitors are predominantly broad-spectrum and non-selective; isoform-selective inhibitors lack human safety data and have only been explored early clinically in oncology, not validated in severe pneumonia populations (132, 133). miRNA antagonists such as anti-miR-155 are still restricted to animal models, hindered by low delivery efficiency, off-target effects and immune risks, with no human clinical trials initiated (134, 135). Collectively, these three targeted strategies lack direct evidence on clinical safety, effective doses and therapeutic windows, representing substantial obstacles to clinical translation.

### 4.3 Systemic regulation strategy

#### 4.3.1 Combined targeted therapy

Based on existing research, a verifiable hypothesis of “PD-1/IL-10 sequential regulation for lung protection” has been proposed for the combined targeted therapy of severe pneumonia-related lung injury: In the acute phase, brief activation of the PD-1 pathway can reduce the release of pro-inflammatory factors such as TNF-α, suppressing the excessive inflammatory response and alleviating early lung injury. Subsequently, during the repair phase, administration of IL-10 can maintain pulmonary immune homeostasis by inducing regulatory T cells and inhibiting chemokines such as monocyte chemoattractant protein-1, achieving a balance between pathogen clearance and tissue repair. Luo et al. (136) assessed the differences in lymphocyte subpopulations between pneumonia and non-pneumonia patients after PD-1/PD-L1 blockade therapy in non-small cell lung cancer patients. They found that in pneumonia patients, PD-1^+^CD8 T cell density significantly increased after treatment, and PD-1/PD-L1 blockade in a pneumonia model exacerbated lung injury, suggesting a protective role of PD-1 in early inflammation suppression. Furthermore, moderate activation of the PD-1/PD-L1 axis induces both macrophages and T cells to secrete IL-10, which synergistically inhibits T cell overactivation and regulates macrophage polarization, thereby alleviating early lung injury while preventing immune-mediated pathogen clearance during the repair phase. Experimental evidence shows that IL-10 can upregulate the expression of PD-1/PD-L1 on immune cell surfaces (137, 138), indicating a positive feedback mechanism between them, further supporting their collaborative regulatory role. Although IL-10 must be used cautiously in the context of infections to prevent persistent pathogen presence, its regulation of T cell function can still maintain pulmonary immune homeostasis, providing a potential intervention target for early immune balance and tissue protection in severe pneumonia-related lung injury (139). Within this theoretical framework, a sequenced intervention strategy based on initiating indicators (e.g., interferon-gamma/IL-10 ratio >3) and transition indicators (e.g., CD8^+^PD-1^+^Tim-3^+^ T cells ≥40%) can be designed (140, 141), combined with short-term use of PD-1 agonists, IL-10 stratified dosing as alternatives, offering a strategy for immune precision regulation in the treatment of severe pneumonia-related lung injury in the future.

In summary, the “PD-1/IL-10 Temporal Regulation for Lung Protection” strategy is an innovative integrated design based on existing target research, and no fully equivalent temporal combination strategy has been developed. Its core value lies in aligning with the pathological characteristics of severe pneumonia-related lung injury—namely, “anti-inflammatory during the acute phase and repair during the later phase”—and constructing a precise temporal immune regulation framework. This approach offers a novel direction for targeted therapies for such diseases.

#### 4.3.2 AI-assisted network pharmacology

The integration of AI with network pharmacology enables a shift from empirical approaches to data-driven, accurate prediction in pneumonia treatment. By constructing target-pathway-drug networks and incorporating differential gene expression and pathway data relevant to pneumonia-induced lung injury (e.g., NF-κB signaling and cell death pathways), AI models can screen multi-target regulatory potential in natural products (e.g., baicalin, curcumin) and predict their synergistic effects with conventional therapies. In clinical settings, machine learning analyses of single-cell sequencing and metabolomics data can support the optimization of personalized treatment strategies. Nevertheless, discrepancies may exist between predicted network pharmacology outcomes and real-world efficacy; ongoing validation and refinement with real-world data are essential.

## 5 Challenges and prospects

### 5.1 Bottlenecks in translational medicine

#### 5.1.1 Limitations of animal models and disease simulation

Animal models remain indispensable for investigating severe pneumonia-associated lung injury, yet many fail to recapitulate the full pathophysiological complexity of human disease, limiting translational yield. Broadly, contemporary systems fall into two categories (142): large‐animal models (e.g., sheep, horses), which offer closer anatomical correspondence to the human lung and permit larger sampling but are confounded by species-specific immune features (such as ruminant-restricted interstitial macrophage subsets), high costs, and limited critical-care expertise. Non-primate animals (such as the macaque model) serve as a major branch of large animal models. With their genetic background and immune system highly homologous to humans, they can accurately simulate core pathological features of severe pneumonia, including diffuse alveolar damage, inflammatory cytokine storms, and hypoxemia. This makes them valuable for translational research. However, their widespread application is limited by high experimental costs, the need for biosafety level 3 laboratory conditions, and stringent ethical reviews (143, 144). And small‐animal models (e.g., mice, rats), which are economical and tractable but constrained by small body size that restricts sampling, substantial variability in drug dosing, and pronounced divergences in immunobiology relative to humans. As a result, interventions that appear efficacious in animals—including selected inflammasome inhibitors and pyroptosis blockers—have underperformed or raised safety concerns in clinical testing. The advent of humanized mouse models (such as angiotensin-converting enzyme 2 humanized mice) has effectively addressed these limitations. Through adenovirus-mediated gene editing, these models can specifically express human viral receptors or immune-related molecules. They exhibit highly humanized pathological features in simulating lung inflammation and cytokine storms induced by viruses such as SARS-CoV-2, and have been successfully applied in screening small-molecule drugs for severe pneumonia and verifying their mechanisms of action. However, humanized mice still face issues such as simplified lung tissue structure and a lack of a complete human immune microenvironment, necessitating complementary validation with other models (145, 146). Meanwhile, three-dimensional alveolar organoids and lung-on-a-chip models have advanced rapidly. Alveolar organoids effectively mimic the 3D structure, cell–cell interaction, barrier function, repair process and inflammatory response of human alveoli, overcoming the drawbacks of conventional models such as species variation, simple structure and lack of physiological microenvironment (147). Lung-on-a-chip further incorporates fluid shear stress, air-blood barrier, oxygen gradient and mechanical strain, enabling dynamic and precise recapitulation of alveolar-capillary barrier injury and inflammation (148). Given the limitations of these models, research must strictly adhere to the FDA’s 3R principles (Replacement, Reduction, and Refinement), which are the core guiding principles for animal experimentation and drug development worldwide. Specifically, *in vitro* experiments should be prioritized as alternatives to animal models, such as human lung cell co-culture systems, alveolar organoids, and lung-on-a-chip technologies, to reduce animal use while enhancing the clinical translational value of experiments. Moreover, human severe pneumonia frequently unfolds in the context of multi-organ dysfunction and comorbid states (e.g., diabetes mellitus, immunodeficiency), whereas many preclinical models emphasize single-pathogen etiologies or isolated inflammatory axes, thereby underrepresenting the multimorbidity and systemic perturbations that critically shape clinical outcomes and complicate extrapolation.

**Figure 3:**
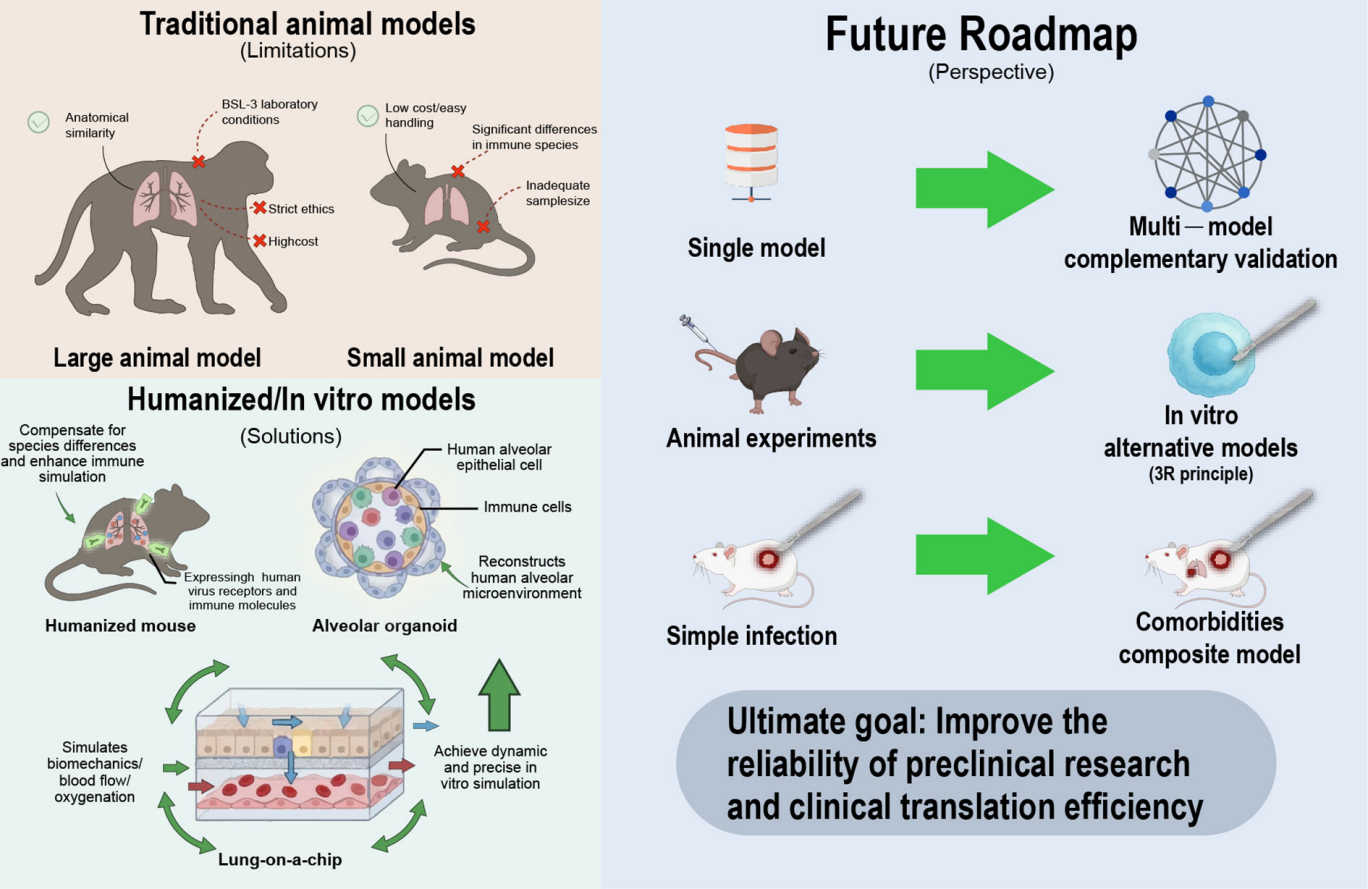
Models of Severe Pneumonia-Associated Lung Injury: Current Limitations and Future Roadmap. This figure summarizes the evolution of preclinical models for severe pneumonia-associated lung injury research. 1. Traditional animal models have obvious limitations: large animals are similar to human lung anatomy but with immune differences, high cost and ethical constraints; small animals are low-cost but with huge immune differences and poor clinical translation. 2. Humanized and in vitro models, including humanized mice, alveolar organoids and lung-on-a-chip, serve as promising solutions. 3. Future directions focus on multi-model validation, in vitro replacement (3R principles) and comorbidity models, to improve preclinical reliability and translation efficiency.

Therefore, future research should adopt a stepwise and progressive technical strategy: shifting from single models to complementary validation with multiple models, promoting the gradual replacement of traditional animal experiments by *in vitro* models based on the 3R principles, and expanding from simple infection models to complex models associated with comorbidities. This will comprehensively improve the reliability of preclinical research and the clinical translation efficiency of intervention strategies for severe pneumonia-related lung injury (see **Figure 3**).

#### 5.1.2 Biomarker specificity and clinical applicability

Current biomarkers, such as the PaO_2_/FiO_2_ ratio and circulating cytokine levels, often lack the specificity needed for early detection and risk stratification. Although novel candidate biomarkers have been identified—ranging from cell death-associated proteins to immune checkpoint molecules—large-scale, multi-center validation remains a critical barrier to clinical implementation. Patient heterogeneity in pathogen type, comorbidity profile, and immune status further complicates the reproducibility and generalizability of biomarker-based diagnostics.

#### 5.1.3 Dual-edged nature of targeted therapies and individual variability

Targeted immunomodulation—exemplified by bidirectional regulation of the PD-1/PD-L1 axis—exerts a “double-edged sword” effect: tempering hyperinflammation can improve outcomes, yet may simultaneously compromise pathogen clearance. Complicating clinical deployment, the optimal parameters for combination regimens—including timing, dosing, sequencing, and patient selection—remain insufficiently defined. These challenges are amplified by substantial interindividual heterogeneity driven by host genetics, baseline immune tone, comorbid conditions, and pathogen diversity, all of which shape highly variable therapeutic responses. Although precision strategies grounded in multi-omics profiling hold promise, truly individualized approaches are still nascent. Overcoming these bottlenecks will require coordinated, multidisciplinary efforts to bridge preclinical and clinical domains—refining disease-relevant animal models, discovering and validating mechanistically anchored biomarkers, and building integrative, multi-omics-based personalization frameworks strengthened by real-world evidence and prospective clinical registries.

### 5.2 Future directions

Advances in severe pneumonia–associated lung injury are poised to center on an integrated, technology-forward paradigm that couples precision diagnosis with next-generation therapeutics and AI-enabled predictive care. First, multi-omics profiling—spanning genomics, proteomics, metabolomics, and other high-dimensional modalities—combined with AI will refine disease subtyping and endotype classification, thereby furnishing a rigorous foundation for individualized treatment planning. Second, therapeutic innovation will be catalyzed by humanized organoid platforms that more faithfully recapitulate human pathophysiology and by optimized nano-targeted delivery systems that enhance tissue specificity and bioavailability, jointly accelerating preclinical-to-clinical translation and improving therapeutic indices. Third, AI-assisted network pharmacology and machine learning applied to large-scale clinical datasets will enable robust prognostic and treatment-response modeling, shifting practice from empiric, reactive management toward prospective, predictive therapy. The synergistic deployment of these capabilities—anchored in multidisciplinary collaboration and strengthened by real-world evidence—promises to overcome current translational bottlenecks, shorten the bench-to-bedside timeline, and deliver safer, more effective, and truly personalized interventions for patients with severe pneumonia-associated lung injury.

## 6 Summary

Severe pneumonia-associated lung injury arises from a dysregulated infection-immunity interplay, driven by complex molecular regulatory networks. This process operates across temporal, spatial, and quantitative dimensions: temporally through distinct disease stages; spatially through inter-organ communication and immune cell distribution; and quantitatively through threshold-dependent mediator activity. We propose a translational research paradigm—Mechanism Analysis-Network Modelling-Precision Intervention—to elucidate pathogenic mechanisms, construct dynamic network-based predictive models, and guide time-specific and node-specific interventions. This framework, integrating mechanistic insights with precision medicine principles, holds promise for overcoming current translational barriers and improving patient outcomes.
